# *E. coli* Fis Protein Insulates the *cbpA* Gene from Uncontrolled Transcription

**DOI:** 10.1371/journal.pgen.1003152

**Published:** 2013-01-17

**Authors:** Kiran Chintakayala, Shivani S. Singh, Amanda E. Rossiter, Rajesh Shahapure, Remus T. Dame, David C. Grainger

**Affiliations:** 1 Institute for Microbiology and Infection, School of Biosciences, University of Birmingham, Birmingham, United Kingdom; 2 Leiden Institute of Chemistry, Gorlaeus Laboratories, Laboratory of Molecular Genetics and Cell Observatory, Leiden University, Leiden, The Netherlands; Institute of Materials Research and Engineering (IMRE), A*STAR, Singapore

## Abstract

The *Escherichia coli* curved DNA binding protein A (CbpA) is a poorly characterised nucleoid associated factor and co-chaperone. It is expressed at high levels as cells enter stationary phase. Using genetics, biochemistry, and genomics, we have examined regulation of, and DNA binding by, CbpA. We show that Fis, the dominant growth-phase nucleoid protein, prevents CbpA expression in growing cells. Regulation by Fis involves an unusual “insulation” mechanism. Thus, Fis protects *cbpA* from the effects of a distal promoter, located in an adjacent gene. In stationary phase, when Fis levels are low, CbpA binds the *E. coli* chromosome with a preference for the intrinsically curved Ter macrodomain. Disruption of the *cbpA* gene prompts dramatic changes in DNA topology. Thus, our work identifies a novel role for Fis and incorporates CbpA into the growing network of factors that mediate bacterial chromosome structure.

## Author Summary

Compaction of chromosomal DNA is a fundamental process that impacts on all aspects of cellular biology. However, our understanding of chromosome organisation in bacteria is poorly developed. Since bacteria are amongst the most abundant living organisms on the planet, this represents a startling gap in our knowledge. Despite our lack of understanding, it has long been known that *Escherichia coli*, and other bacteria, radically re-model their chromosomes in response to environmental stress. This is most notable during periods of starvation, when the *E. coli* chromosome is super compacted. In dissecting the molecular mechanisms that control this phenomenon, we have found that regulatory cross-talk between DNA–organising proteins plays an essential role. Thus, the major DNA folding protein from growing *E. coli* inhibits production of the major chromosome organisers in starved cells. Our findings illustrate the highly dynamic nature of bacterial chromosomes. Thus, DNA topology, gene transcription, and chromosome folding proteins entwine to create a web of interactions that define the properties of the chromosome.

## Introduction

Bacterial chromosomes are organised into a nucleoid by an integrated network of supercoiling, transcription and nucleoid associated DNA binding proteins [1]. This network is highly dynamic and responsive to changes in the extracellular environment. In *Escherichia coli*, a particularly notable change in nucleoid structure occurs when cells are starved. Namely, the nucleoid adopts a super compact conformation that is believed to protect the genome. These changes in nucleoid structure coincide with changes in supercoiling [2], transcription [3] and the available pool of nucleoid proteins [4]. Strikingly, only two nucleoid proteins are specifically up-regulated as cells approach stationary phase; the DNA binding protein from starved cells (Dps) and Curved DNA binding protein A (CbpA) [4]. The Dps protein has been studied for decades and has well characterised DNA binding, compaction and protection properties [5]–[7]. In growing cells expression of Dps is blocked by Fis, the major growth phase nucleoid protein [8]. In sharp contrast to Dps, the regulation and DNA binding properties of CbpA have hardly been studied.

The CbpA protein was first isolated as a factor present in crude *E. coli* cell extracts that preferentially bound curved DNA *in vitro* [9]. The affinity of CbpA for DNA is similar to that observed for other nucleoid associated proteins [10]. CbpA consists of an N-terminal J-domain separated from two C-terminal domains (CTDI and II) by a flexible linker [11]. The J-domain interacts with the modulator protein CbpM, which inhibits CbpA co-chaperone activity and DNA recognition [12]–[14]. DNA binding activity locates to the linker-CTDI region and CTDII mediates dimerisation [11], [15]. Transcription of *cbpA* initiates from overlapping promoters referred to as P1 and P2 [16]. Most *cbpA* transcription is driven by the σ^38^ dependent P2 promoter with σ^70^ dependent P1 making only a small contribution [16]. Consistent with this, CbpA accumulates in starved *E. coli*, reaching 15,000 copies per cell after two days [4]. In contrast to Dps, which is uniformly distributed within the nucleoid, CbpA forms nucleoid associated foci [17]. Nothing is known about the function of CbpA in starved *E. coli* cells.

In this work we sought to better understand the regulation and function of CbpA. We show that Fis plays a crucial role in preventing CbpA expression in growing cells. Hence, Fis binds to DNA target sites in the *cbpA* regulatory region. When bound to these sites Fis prevents *cbpA* being transcribed from an aberrant promoter, located within the coding sequence of an adjacent gene. In starved cells, when *cbpA* transcription is induced by σ^38^, CbpA binds pervasively across the *E. coli* genome with a bias to the intrinsically curved Ter macrodomain. Disruption of the *cbpA* gene prompts dramatic changes in DNA supercoiling.

## Results

### CbpA expression is uncoupled from growth-phase in a *fis* mutant

**Figure 1:**
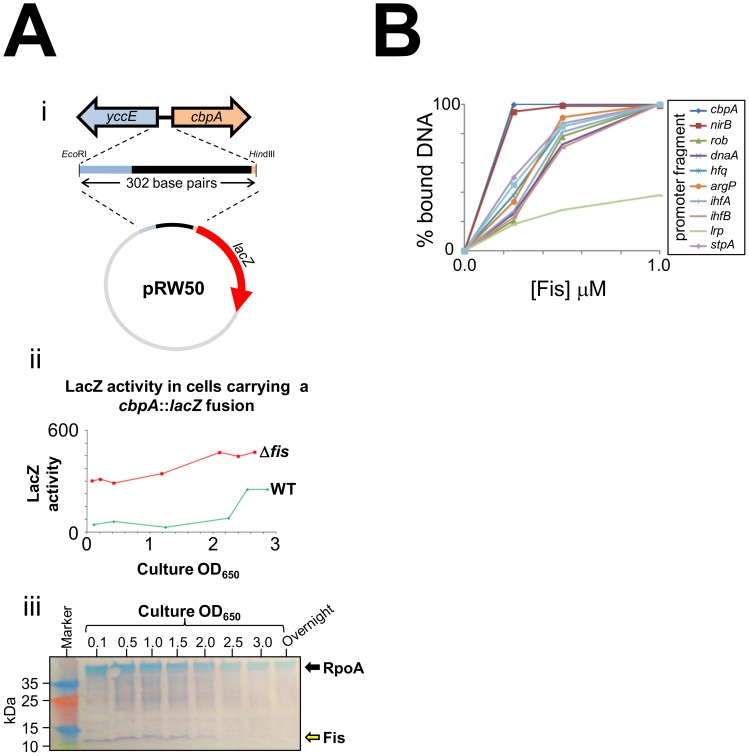
CbpA expression is uncoupled from growth-phase in a *fis* mutant. (A) Activity of a *cbpA*::*lacZ* fusion in different growth-phases. i) Schematic representation of the *cbpA*::*lacZ* fusion. A 302 base pair DNA fragment, encompassing the *cbpA* start codon, the entire *cbpA*-*yccE* intergenic region, and a portion of the *yccE* gene, was cloned upstream of *lacZ* in the low copy number *lacZ* reporter plasmid pRW50. ii) The graph shows LacZ activity calculated for either wild type or Δ*fis* cells carrying the plasmid construct shown in panel i). iii) Western blot showing Fis levels in *E. coli* K-12 throughout growth and in overnight cultures. The blots were also probed with antibodies against RpoA as a control. (B) Fis has a high affinity for the *cbpA* regulatory region. The graph illustrates the binding of Fis to different DNA fragments (see key). The raw EMSA data are shown in Figure S1.

As *E. coli* cells approach stationary phase they induce expression of two nucleoid proteins, Dps and CbpA. Previously, we found that Dps expression in growing *E. coli* cells is repressed by Fis [8]. Thus, we wondered if Fis may also control production of CbpA. In an initial experiment we cloned a 302 base pair DNA fragment, containing the entire *cbpA* regulatory region and part of the adjacent *yccE* gene, upstream of *lacZ* (illustrated schematically in Figure 1Ai). We then measured LacZ activity in WT JCB387 cells, carrying this fusion, throughout growth. We observed basal levels of LacZ activity until the onset of stationary phase at which point LacZ activity increased (green line in Figure 1Aii). Strikingly, in JCB3871Δ*fis* cells, LacZ activity was high throughout the time course (red line in Figure 1Aii). In complementary western blotting experiments we measured Fis levels in *E. coli* cells at different stages of growth (Figure 1Aiii). Fis levels were inversely correlated with *cbpA* induction. Intrigued by this observation we measured Fis binding to the *cbpA* regulatory DNA using Electrophoretic Mobility Shift Assays (EMSA). For comparison, we also tested DNA fragments that drive expression of other nucleoid proteins and the *nirB* promoter, which has a high affinity for Fis [18]. The raw EMSA data are shown in Figure S1 and a quantification of the experiment is shown in Figure 1B. The data show that Fis binds particularly tightly to the *cbpA* regulatory region. Note that Fis forms three distinct complexes with the *cbpA* regulatory DNA, suggesting three separate Fis binding sites (Figure S1).

### Location of Fis binding sites at the *cbpA* regulatory region

**Figure 2:**
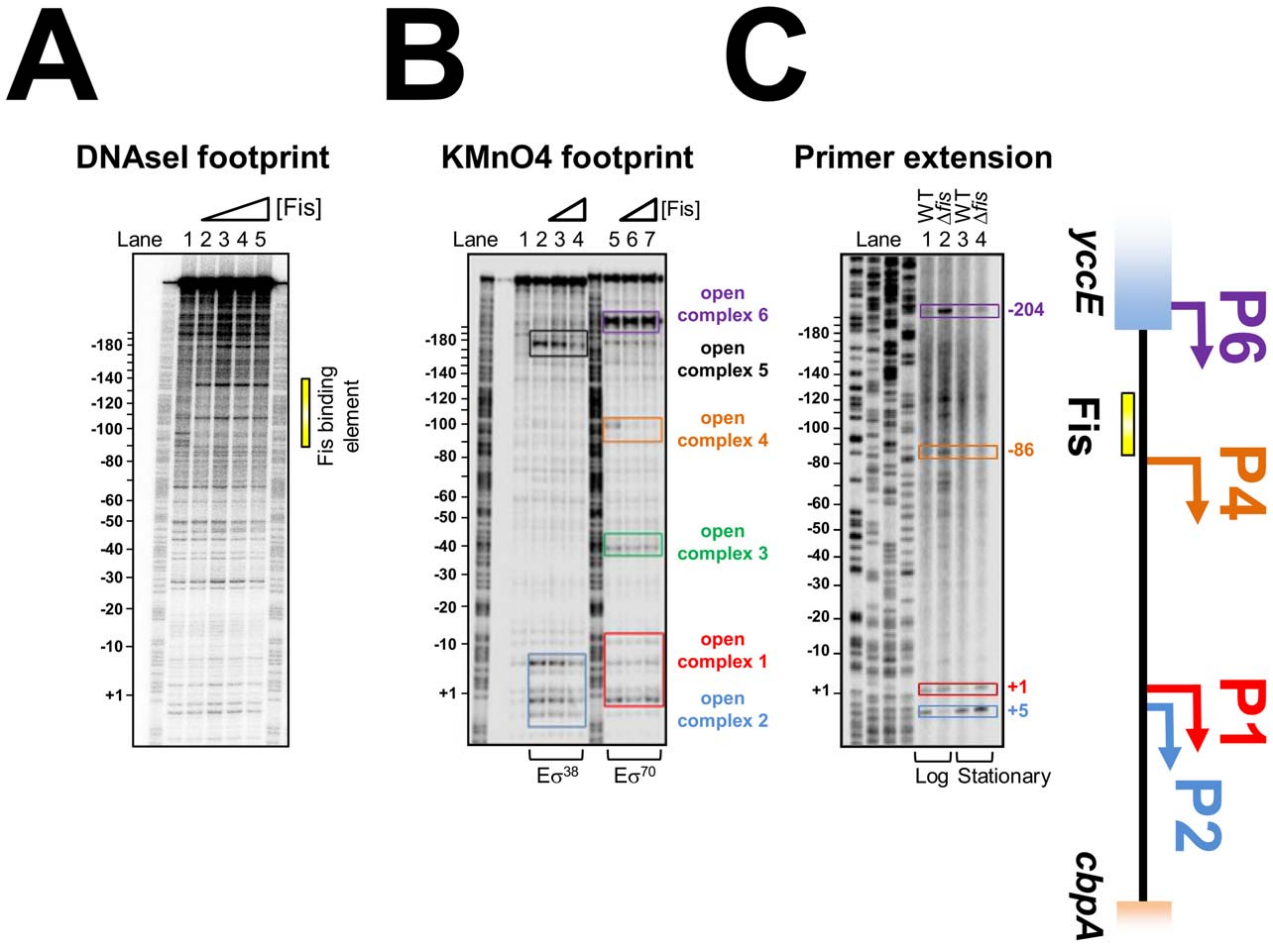
Location of Fis, RNA polymerase, and transcription start sites at the *cbpA* regulatory region. (A) Fis binds to a 55 base pair DNA region upstream of *cbpA*. DNAseI footprinting analysis of Fis binding to the *cbpA* regulatory region. The image shows a scan of a dried polyacrylamide sequencing gel on which DNase I digestion of the 302 base pair *cbpA* fragment (∼20 nM) was compared in the presence and absence of 250 nM, 500 nM or 1 µM Fis. The 55 base pair element bound by Fis is highlighted by a yellow bar. (B) Location of RNA polymerase binding sites *in vitro*. Results of a KMnO_4_ footprinting experiment to detect open complex formation by either σ^38^ or σ^70^ associated RNA polymerase (400 nM) in the presence and absence of Fis (250 or 500 nM). Open complexes are highlighted by coloured boxes and are numbered. (C) Location of *cbpA* transcription start sites *in vivo*. The gel shows a *cbpA* mRNA primer extension analysis. The location of transcription start sites are given with respect to the *cbpA* P1 promoter (+1). Bands corresponding to transcripts that initiate from the P1 and P2 promoters are highlighted in red and blue respectively. Two further transcript start sites, observed only in growing JCB3871Δ*fis* cells, are highlighted in orange and purple. A schematic of the *cbpA* regulatory region is shown to the right of the figure.

Fis binds to a 15-base-pair AT-rich DNA target that is highly degenerate. A common feature of many Fis sites is a G at position 1 and a C at position 15; however even these features are not universally conserved [19]–[21]. Thus, DNAse I footprinting was used to locate Fis bound at the *cbpA* regulatory region. The full sequence of the 302 base pair DNA fragment is shown in Figure S2. The previously characterised *cbpA* P1 and P2 promoters are highlighted. Throughout this work, all numbering is with respect to the P1 promoter. The results show that Fis binds a DNA element between 90 and 145 base pairs upstream of the *cbpA* P1 transcript start (Figure 2A). Since Fis binds to a 15 base pair recognition sequence the large 55 base pair footprint most likely contains three Fis binding sites, as observed in our EMSA analysis (Figure S1). Scrutiny of the DNA sequence corresponding to the region bound by Fis identified one match to the canonical Fis binding sequence (i.e. containing a G at position 1 and a C at position 15). This site is centred 101 base pairs upstream of the P1 transcription start (Figure S2) and can be disrupted by altering the key positions in the Fis recognition sequence. Thus, the *cbpA*-108C-94G DNA fragment has a greatly reduced affinity for Fis in EMSA assays (Figure S3). We presume that adjacent DNA sites for Fis in this region must have an atypical sequence. This is not exceptional and similar observations have been made for Fis binding sites at the *rrn* promoters [20].

### Location of RNA polymerase binding sites at the *cbpA* regulatory region

As a first step to understanding control of *cbpA* by Fis we utilised KMnO_4_ footprinting that detects DNA melting around transcription start sites. Thus we were able to measure RNA polymerase binding *in vitro*, to the *cbpA* regulatory region, in the presence and absence of Fis. Recall that *cbpA* transcription can be stimulated by σ^70^ or σ^38^ associated RNA polymerase. The results in the absence of Fis, illustrated in Figure 2B, show different patterns of σ^38^ (lane 2) and σ^70^ (lane 5) dependent DNA opening. As expected, our analysis identified DNA melting at the known P1 and P2 promoters (highlighted by red and blue boxes in Figure 2B). Surprisingly, we also observed σ^70^ dependent DNA opening at three further locations (highlighted by green, orange and purple boxes in Figure 2B) and one additional σ^38^ dependent DNA opening event (highlighted by a black box in Figure 2B). Given the large effect of Fis on *cbpA* expression *in vivo* (Figure 1Aii), we were surprised that addition of Fis to the KMnO_4_ footprinting reaction had minor effects (lanes 3–4 and 6–7). Thus, Fis only inhibited DNA untwisting at position −100 (open complex 4) which overlaps the Fis binding element.

### Identification of *cbpA* transcription start sites *in vivo*

To aid interpretation of the *in vitro* KMnO_4_ footprinting analysis we conducted *in vivo* mRNA primer extension experiments. This enabled us to identify *cbpA* transcript start sites, used in the presence and absence of Fis, in growing and stationary phase cells. The results of this analysis are shown in Figure 2C. In WT cells we observed only two *cbpA* mRNA primer extension products. As expected these correspond to the P1 and P2 promoters (see lanes 1 and 3 in Figure 2C). The Δ*fis* mutation had little effect on transcription start site selection in stationary phase cells (see lane 4 in Figure 2C). However, the Δ*fis* mutation had a dramatic effect in growing cells (see lane 2 in Figure 2C). Thus, in growing Δ*fis* cells, the most abundant *cbpA* transcript did not originate from either the P1 or P2 promoter. Rather, it initiated form a site located 204 base pairs upstream of the P1 promoter in the *yccE* gene (highlighted in purple in Figure 2C). This transcription start site, labelled P6 in the schematic, aligns precisely with open complex 6 in the KMnO_4_ footprinting experiments (compare Figure 2B and 2C). An additional transcription start site, which we refer to as P4, aligns with open complex 4 (see orange highlighting in Figure 2B and 2C). Note that additional primer extension products in lane 2 of Figure 2C, which are also close to open complex 4, are likely due to degradation of longer transcripts. To confirm that we correctly identified the P4 promoter, we created a P4::*lacZ* fusion and investigated the effect of mutating the proposed −10 element (Figure S4A). The data show that the P4 promoter drives LacZ expression and that transcription from the P4 promoter increases when the proposed −10 element is improved (Figure S4B). However, overall P4 makes only a small contribution to *cbpA* transcription.

### Fis represses transcription from the *cbpA* P4 and P6 promoters *in vitro*

**Figure 3:**
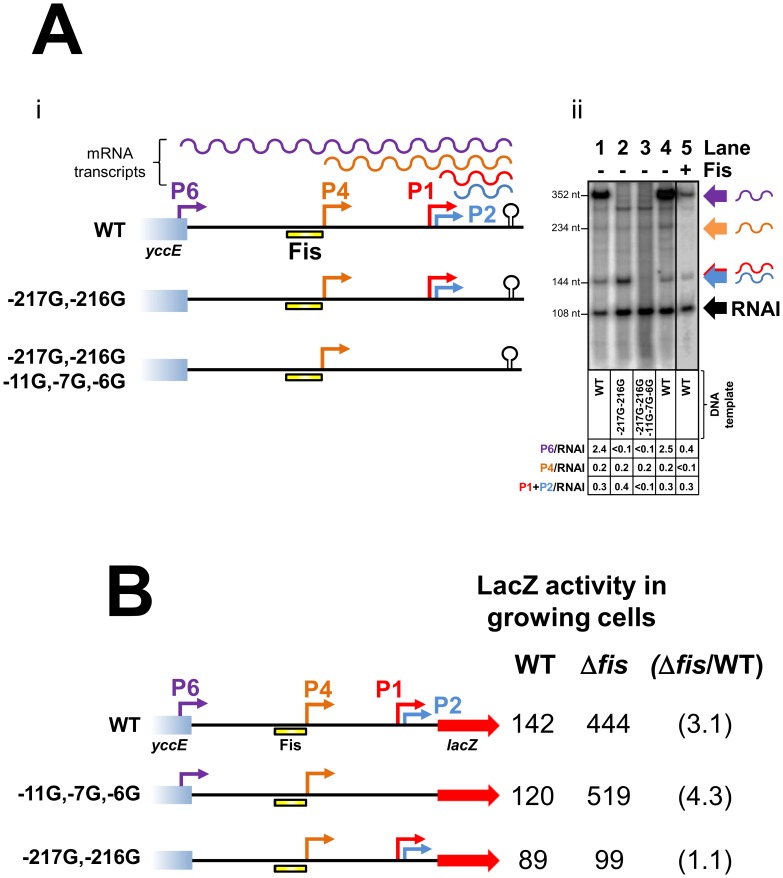
Repression of transcription from the P4 and P6 promoters by Fis. (A) Repression by Fis *in vitro*. i) Schematic of the *cbpA* regulatory region DNA used for *in vitro* transcription assays. Promoters are shown by coloured arrows and the λ*oop* transcription terminator is shown by a black “lollipop”. The Fis binding element is shown as a yellow bar. Different mRNA transcripts are shown by coloured wavy lines. ii) mRNA transcripts generated by a combination of σ^70^ and σ^38^ associated RNA polymerase (400 nM each) in the presence and absence of Fis (500 nM). Transcripts are labelled according to the scheme in panel i). (B) Repression by Fis *in vivo* requires P6 and the Fis binding element. Different *cbpA*::*lacZ* fusions are illustrated. Promoters are shown by coloured arrows. The Fis binding element is shown as a yellow bar. LacZ activity values from growing JCB387 and JCB3871Δ*fis* cells are given adjacent to each promoter::*lacZ* fusion. The fold repression by Fis is shown in parenthesis.

To investigate further the effect of Fis on each *cbpA* promoter we used *in vitro* transcription assays. The 302 base pair *cbpA* regulatory DNA fragment was cloned upstream of the factor-independent λ*oop* transcription terminator in plasmid pSR. The different mRNA products expected to be produced using this DNA template are illustrated in Figure 3Ai. We also created two derivatives of this construct lacking either the P6 promoter or a combination of the P6, P2 and P1 promoters. The different promoters were disrupted by making mutations in the promoter −10 element. Thus, the −217G–216G mutation negates the P6 promoter, the −11G mutation disrupts the P2 promoter and the −7G–6G mutation abolishes transcription from P1 (Figure 3Ai). Figure 3Aii shows the results of *in vitro* transcription assays with the different DNA templates. As expected, using the wild type DNA template, a mixture of σ^38^ and σ^70^ associated RNA polymerase stimulated production of transcripts corresponding to the P1, P2, P4 and P6 promoters (Figure 3Aii lane 1). The −216G–217G mutation abolished transcription from the P6 promoter (Figure 3Aii lane 2). Similarly, mutations −6G,−7G and −11G, which disrupt the P1 and P2–10 hexamers respectively, abolish transcription from the P1 and P2 promoters (Figure 3Aii lane 3). Note that, the P1 and P2 transcripts differ in length by only 5 nucleotides and cannot be resolved in this experiment. Lanes 4 and 5 of Figure 3Aii show the pattern of transcripts generated in the absence and presence of Fis respectively. As expected, Fis greatly reduced transcription from the P6 and P4 promoters. Conversely, Fis had only a minor effect on the P1 and P2 promoters. Note that the small RNAI transcript, from the pSR replication origin, acts as an internal control.

### Fis has no effect on *cbpA* expression in the absence of P6

Taken together our data suggest that Fis prevents CbpA expression in growing cells primarily by silencing the strong P6 promoter. To confirm this we designed *cbpA::lacZ* fusions lacking either P1 and P2, or P6, due to mutations in the promoter −10 hexamer. Our hypothesis was that disruption of the P6 promoter would negate the effect of Fis whereas disruption of P1 and P2 would not. The different DNA fragments, fused to *lacZ*, are illustrated in Figure 3B alongside LacZ activity data from growing JCB387 or JCB3871Δ*fis* cells. As expected, LacZ expression from the wild type *cbpA::lacZ* fusion increased in JCB3871Δ*fis* cells. An almost identical result was obtained using the −11G,−7G,−6G fragment lacking P1 and P2. Hence, Fis does not exert its effect via the P1 and P2 promoters. The DNA fragment lacking the P6 promoter, due to the −217G and −216G mutations, stimulated low levels of LacZ expression that *did not* increase in JCB3871Δ*fis* cells.

### Deletion of the Fis binding region leads to de-repression of P6

**Figure 4:**
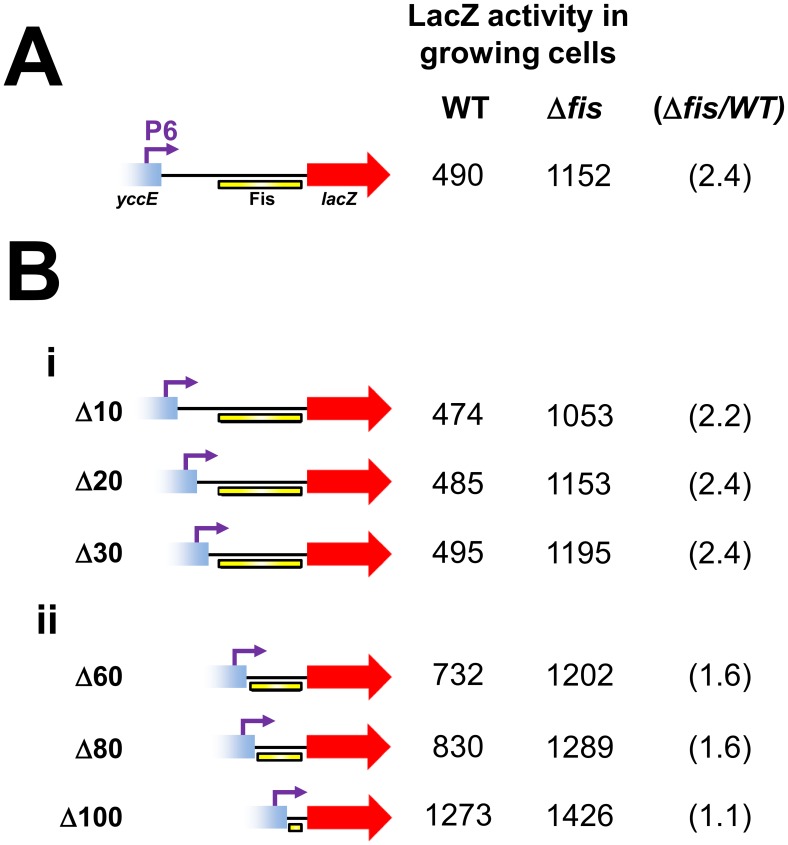
Deletion analysis of the Fis binding region. (A) The P6::*lacZ* fusion. The P6 promoter is shown as a purple arrow. The Fis binding element is shown as a yellow bar. LacZ activity values from growing JCB387 and JCB3871Δ*fis* cells are given adjacent to the illustrated promoter::*lacZ* fusion. The fold repression by Fis is shown in parenthesis. (B) Deletion analysis of the P6*::lacZ* fusion. Panel i) shows deletions (Δ) of 10, 20 or 30 base pairs introduced downstream of the *cbpA* P6 transcription start site. These deletions do not disrupt the region bound by Fis. Rather, they move the region bound by Fis closer to the P6 promoter. LacZ activity values from growing JCB387 and JCB3871Δ*fis* cells are given adjacent to each promoter::*lacZ* fusion. The fold repression by Fis is shown in parenthesis. Panel ii) shows deletions of (Δ) of 60, 800 or 100 base pairs introduced downstream of the *cbpA* P6 transcription start site. The Δ60, Δ80 and Δ100 deletions sequentially degrade the region bound by Fis. LacZ activity values from growing JCB387 and JCB3871Δ*fis* cells are given adjacent to each promoter::*lacZ* fusion. The fold repression by Fis is shown in parenthesis.

To confirm that the Fis binding region was responsible for mediating repression of the P6 promoter we generated a series of P6*::lacZ* fusions containing nested deletions downstream of the P6 transcription start site. The different DNA fragments, fused to *lacZ*, are illustrated in Figure 4 alongside LacZ activity data from growing JCB387 or JCB3871Δ*fis* cells. The starting P6*::lacZ* fusion, containing the P6 promoter and full Fis binding region, stimulated *lacZ* expression that increased in cells lacking *fis* (Figure 4A). Deletions of between 10 and 30 base pairs (Δ10, Δ20 and Δ30) sequentially place the Fis binding element closer to the P6 promoter. These deletions had little effect on either LacZ expression or repression by Fis (Figure 4Bi). Conversely, larger 60, 80 and 100 base pair deletions (Δ60, Δ80, Δ100), which sequentially degrade the Fis binding element, lead to stepwise increases in LacZ expression and a reduction in repression by Fis (Figure 4Bii). Thus, our data are consistent with Fis “protecting” *cbpA* from the effects of the P6 promoter by interacting with the Fis binding region.

### Binding of Fis and RNA polymerase to the *cbpA* chromosomal locus *in vivo*

We next sought support for our model by re-evaluating published data from three independent chromatin immunoprecipitation (ChIP) studies of Fis binding across the *E. coli* chromosome [22]–[24]. We also scrutinised our own unpublished mRNA deep sequencing (RNA-seq) data from *E. coli* cells at different stages of growth. Inspection of the ChIP data revealed that DNA upstream of *cbpA* regulatory region was a target for Fis in all three ChIP studies. Strikingly, the single nucleotide resolution mapping of Fis binding performed by Kahramanoglou *et al* [24] identified exactly the Fis binding site defined by our *in vitro* footprinting analysis (Figure S5). Examination of RNA-seq data revealed that no transcripts originate from the *cbpA* P6 promoter during rapid growth when Fis levels are high (Figure S5). Conversely, in starved *E. coli* cells that contain low levels of Fis, transcription from the P6 promoter was evident (Figure S5).

### The *cbpA* Fis binding region reduces LacZ expression from non-canonical promoters

We reasoned that we should be able to “transplant” the repressive effect of the *cbpA* Fis binding region to unrelated promoters. Furthermore, we expected that the Fis binding region would only inhibit promoters active during periods of growth, when Fis levels are high. Thus, we selected four promoter regions with different regulatory properties. Three of the promoters that we selected (*gal*P1, *ynfE* and *yeaR*) are σ^70^ dependent promoters active in growing cells. Transcription from *gal*P1 is stimulated by CRP, *ynfE* is FNR dependent and *yeaR* transcription requires NarL [25]–[27]. The fourth promoter that we selected (*aer*) is σ^28^ dependent and active during periods of starvation [28]. We compared LacZ expression from each promoter either in the presence or absence of the *cbpA* Fis binding element. When present the Fis binding region was placed between the promoter transcription start site and the *lacZ* start codon. The data show that the Fis binding element from the *cbpA* regulatory region repressed LacZ expression from all three “growth phase” promoters. Conversely, the *aer* promoter, which is active during periods of starvation, was not repressed (Table 1).

**Table 1:** LacZ expression from different promoter::*lacZ* fusions in the presence and the absence of the *cbpA* Fis binding region.

	LacZ activity	
Promoter	− *cbpA* Fis binding region	+ *cbpA* Fis binding region
***gal*** **P1**	602	398
***ynfE***	298	150
***yeaR***	51	31
***aer***	158	251

The table shows **β**-galactosidase activities measured in *Escherichia coli* JCB387 cells, containing pRW50 carrying different promoter derivatives. Where present the *cbpA* Fis binding region is located between the *lacZ* start codon and the promoter transcription start site. Each data point is the average from at least three independent experiments with a standard deviation of <10%.

### CbpA binding across the chromosome of starved *E. coli*

**Figure 5:**
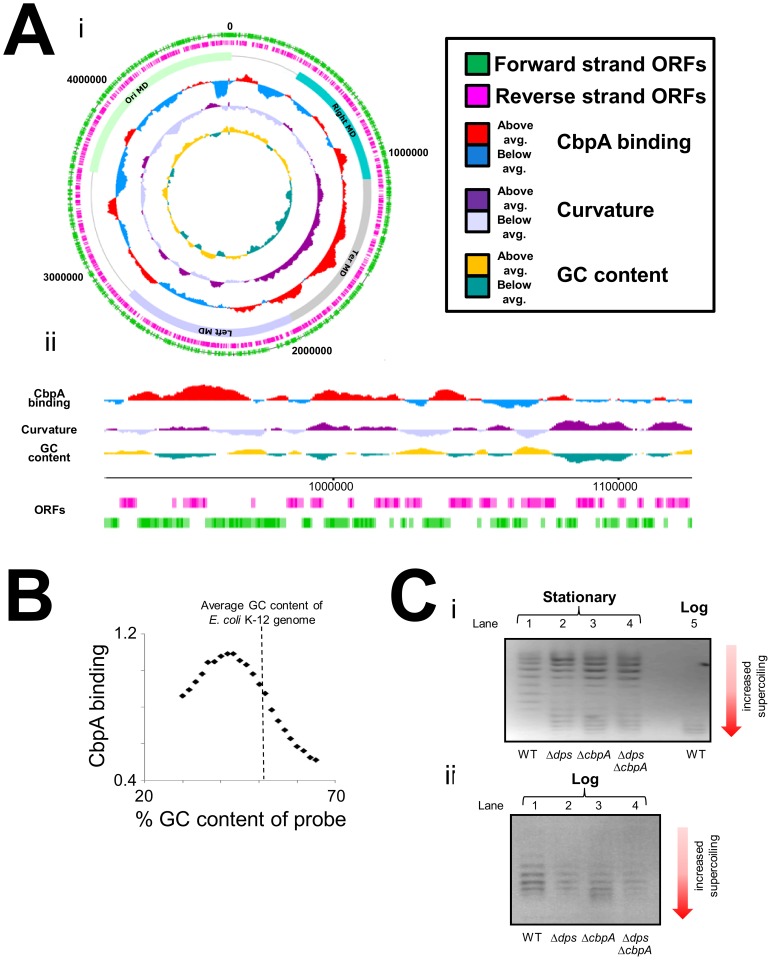
Chromosome-wide distribution of CbpA in starved *E. coli*. (A) Distribution of CbpA across the *E. coli* chromosome. i) Genome-wide view of CbpA binding in starved MC108 cells. The figure shows ChIP-chip data for CbpA binding plotted against features of the *E. coli* genome in the form of a genome atlas. The data have been averaged across a 100,000 base pair window. The four chromosomal macrodomains (MD) are labelled ii) CbpA ChIP-chip data for a small section of the *E. coli* chromosome. The data have been averaged across a 10,000 base pair window. (B) Relationship between CbpA binding and DNA GC content. The graph shows the average CbpA binding signal plotted against the GC content of probes on the DNA microarray. The average GC content of the *E. coli* K-12 chromosome is shown by a dashed line. Very GC rich and GC poor probes were excluded during microarray design and are thus absent. (C) Effect of CbpA on DNA supercoiling *in vivo*. Panel i) shows an image of a 1% (v/v) agarose gel containing 2.5 µg/ml chloroquine. Plasmids were isolated from different genetic backgrounds and different stages of growth as indicated. Panel ii) also shows an image of a 1% (v/v) agarose gel containing 2.5 µg/ml chloroquine. Plasmids were isolated from rapidly dividing cells.

We next turned our attention to the DNA binding properties of CbpA in starved *E. coli*. To investigate DNA binding *in vivo* we utilised chromatin immunoprecipitation and DNA microarrays (ChIP-chip). In a preliminary analysis we compared CbpA binding in wild type *E. coli* BW27784 and the *cbpM* derivative MC108. The data show similar patterns of CbpA binding across the *E. coli* chromosome in both cases (Figure S6). Recall that CbpM is known to interfere with DNA binding by CbpA. Hence, *in vivo* sequence requirements for CbpA-DNA interactions were determined using ChIP-chip data generated in the absence of CbpM. Because CbpA was isolated on the basis of its propensity to bind curved DNA *in vitro* we aligned the CbpA ChIP-chip profile with profiles of predicted DNA curvature [29] and DNA GC content. Figure 5Ai gives an overview of the alignments for the whole genome. Figure 5Aii provides a more detailed view for a smaller segment of the chromosome. Two characteristics of CbpA binding are apparent. First, CbpA binding is biased towards the Ter macrodomain (Figure 5Ai). Second, there is a positive correlation between CbpA binding and DNA curvature (Figure 5Ai and 5Aii). This pattern of DNA binding was also confirmed *in vitro* for selected targets (Figure S7). To better ascertain the relationship between DNA sequence and CbpA binding we grouped all probes on the DNA microarray according to their percentage GC content. For each group of probes we then calculated the mean CbpA binding signal. The results of this analysis confirm that CbpA binding is greatly reduced at GC-rich DNA sequences (Figure 5B). Optimal CbpA binding was observed at DNA with a GC content of 43%. The average GC content of the *E. coli* genome is 51%. We note that, whilst CbpA seldom binds to regions that are not intrinsically curved, not all regions of predicted curvature are bound by CbpA (Figure 5Aii). This is likely due to competition with other proteins that recognise curved DNA. Similarly, the *in silico* DNA curvature predictions may not hold true at all locations *in vivo*.

### CbpA influences DNA topology in stationary phase *E. coli* cells

Numerous studies have shown that entry to stationary phase results in a marked decrease in DNA supercoiling [2], [30], [31]. Thus, we investigated the effect of CbpA, and as a control Dps, on DNA topology *in vivo* using a reporter plasmid. After extraction from three day old cultures plasmid topoisomers were separated on a 1% agarose gel containing 2.5 µg/ml chloroquine. A sample of plasmid DNA isolated from log phase cells was also analysed as a control. As expected, the plasmid isolated from growing cells was considerably more supercoiled than the plasmid isolated from starved cells (Figure 5Ci, compare lanes 1 and 5). Remarkably, starved cells lacking *dps* or *cbpA* yielded a split distribution of plasmid topoisomers. Some plasmids were highly supercoiled with similar topology to plasmids isolated from growing cells (compare lanes 2, 3 and 5). Other plasmids in the sample were relaxed, as seen for starved cells (compare lanes 1–3). Plasmids isolated from the Δ*dps*Δ*cbpA* strain had similar topology to the plasmids isolated from the individual gene knockout strains (compare lanes 2–4). These effects were specific to plasmids isolated from starved cells; plasmid topoisomers obtained from growing cultures of each strain did not significantly differ (Figure 5Cii).

## Discussion

### Mechanism of *cbpA* repression by Fis during rapid growth

**Figure 6:**
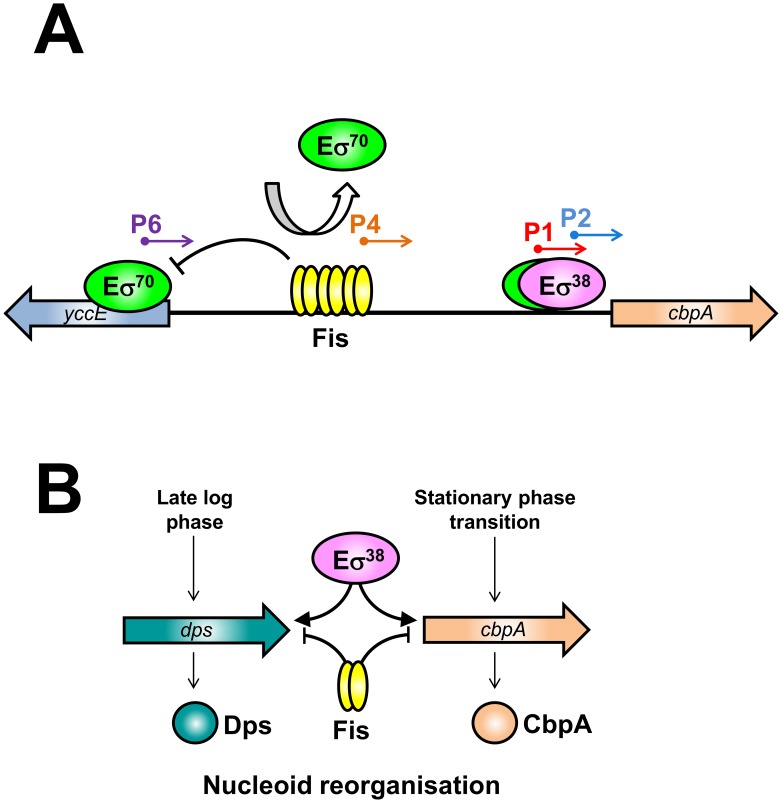
The nucleoid protein response to starvation in *E. coli*. (A) Model of *cbpA* regulation by Fis. (B) Model for staged induction of *cbpA* and *dps*.

In wild type *E. coli* cells *cbpA* transcription initiates from the P1 and P2 promoters with the σ^38^ dependent P2 promoter being dominant [16], [32] (Figure 2C). However, during rapid growth, Fis is required to prevent uncontrolled transcription of *cbpA* (Figure 1Aii). This uncontrolled transcription is primarily driven by the P6 promoter, which is located in the *yccE* gene, more than 250 base pairs upstream of *cbpA*. Whilst Fis prevents transcription from P6 *in vivo* (Figure 2C, Figure 3B and Figure 4) and *in vitro* (Figure 3A) Fis appears unable to prevent RNA polymerase binding at the P6 promoter (Figure 2B). This is not surprising since the DNA element bound by Fis is located ∼60 base pairs downstream of the P6 transcription start. We conclude that Fis must either act as a “road block”, to prevent transcription elongation, or prevent RNA polymerase escape from the P6 promoter. We currently favour the latter model since we were unable to detect “road blocked” transcripts in our *in vitro* transcription assay (Figure 3A). Note that Fis also inhibits transcription from the weak P4 promoter (Figure 2C, Figure 3A and Figure S4). In this case the P4 −10 element is embedded within the primary Fis binding sequence (Figure S4). Hence, Fis binding to this site prevents RNA polymerase association with the P4 promoter (Figure 2B lanes 5–7). Our model for Fis regulation of *cbpA* is outlined in Figure 6A. This regulatory mechanism is salient since it is becoming increasingly apparent that bacterial chromosomes contain many intragenic promoters [33]–[35]. Thus, mechanisms must exist to ensure that these promoters do not adversely affect transcription of neighbouring operons. We speculate that Fis, and other nucleoid proteins, may serve such a purpose.

### Role of Fis in controlling nucleoid re-organisation on transition to stationary phase

Taken together with previous work our results provide a detailed molecular explanation for the phenomenon of nucleoid reorganisation that occurs in starved *E. coli* cells [36]. We propose that regulatory crosstalk between nucleoid proteins plays a pivotal role. In our model, Fis sits at the fulcrum of the regulatory process by binding low affinity elements overlapping the *dps* promoter [8] (to inhibit σ^70^ dependent transcription) and by binding high affinity sites in the *cbpA* regulatory region (Figure 1) (to insulate *cbpA* from σ^70^ dependent transcription). As cells divide, and Fis levels decrease, the different affinity of Fis for the *dps* and *cbpA* regulatory regions contributes to staged induction of *dps* and *cbpA* (Figure 6B). Interestingly, despite their similar DNA binding properties, CbpA and Dps both have distinct stress response functions. Thus, CbpA can function as a co-chaperone, by virtue of its N-terminal J-domain, whilst Dps has a bacterioferritin like fold and can sequester Fe^2+^ ions [7], [9]. We speculate that, to fully understand the function of CbpA and Dps in stationary phase, the relationship between their different activities will need to be unravelled.

## Materials and Methods

### Strains, plasmids, and oligonucleotides

Bacterial strains, plasmids and oligonucleotide sequences are listed in Table S1. All *cbpA* regulatory region sequences are numbered with respect to the P1 transcription start point (+1) and with upstream and downstream locations denoted by ‘−’ and ‘+’ prefixes respectively.

### β-galactosidase assays

Activities are shown in Miller units [37] and are the average of three or more independent experiments with a standard deviation of <10%. Background LacZ activity values, generated from cells carrying a “promoterless” pRW50, were subtracted. Cells were grown aerobically, at 37°C, in LB media. Assays were performed using either JCB387 or the derivative JCB3871Δ*fis*.

### mRNA primer extension assays

Transcript start sites were mapped by primer extension, as described in Lloyd *et al*. [38], using RNA purified from strains carrying the 302 base pair *cbpA* regulatory DNA fragment cloned in pRW50. The 5′ end-labelled primer D49724, which anneals downstream of the *Hin*dIII site in pRW50 was used in all experiments. Primer extension products were analysed on denaturing 6% polyacrylamide gels, calibrated with arbitrary sequencing reactions, and visualized using a Fuji phosphor screen and Bio-Rad Molecular Imager FX.

### Protein purification, *in vitro* DNA binding, and *in vitro* transcription assays

CbpA and derivatives were all purified as described [15]. Fis and RNA polymerase were prepared as described previously [8]. EMSA, DNAseI and KMnO4 footprinting with Fis and/or RNA polymerase are described by Grainger *et al*. [8]. The *in vitro* transcription experiments were performed as described [8] using the system of Kolb *et al.* [39]. Protein and DNA concentrations used for all *in vitro* experiments are provided in the figure legends.

### Chromatin immunoprecipitation and DNA microarray analysis

Chromatin Immunoprecipitation was done exactly as described previously [40]. Formaldehyde crosslinked nucleoprotein obtained from stationary phase BW27784 or MC108 cells was fragmented by sonication and CbpA-DNA complexes were precipitated using a rabbit polyclonal antibody against CbpA. A control mock immunoprecipitation (from which anti-CbpA was omitted) was done in parallel. The “plus and minus antibody” DNA samples were then labelled with Cy5 and Cy3 respectively before being mixed and hybridised to a 43,450 feature DNA microarray (Oxford Gene Technology). After hybridisation, washing and scanning the Cy5 and Cy3 signal was calculated for each probe on the array (Table S2). The MC108 experiment was done in duplicate, and an average Cy5/Cy3 ratio was used for further analysis (referred to as the CbpA binding signal). The images shown in Figure 4 were generated using DNA plotter software [41]. To facilitate detailed inspection of the CbpA ChIP-chip data a file that can be loaded into DNA plotter or the Artemis genome browser is provided in the supplementary material (Table S3). These data should be loaded into the software as a graph after first installing the *E. coli* K-12 genome sequence (provided as a genbank file in Table S4).

### Analysis of DNA curvature and GC content

The DNA curvature analysis of the *E. coli* chromosome was done using the CURVATURE software package [42] exactly as described previously [29]. The DNA GC profile was calculated using the internal graph function in DNA plotter. We note that low GC content is not an absolute indicator of increased DNA curvature on a local scale of a few base pairs. However, for the large segments of DNA considered here, there is a clear inverse correlation between GC content and DNA curvature.

### Chloroquine gel electrophoresis

We monitored superhelicity of plasmid pJ204 in strain BW27784 and the Δ*dps*, Δc*bpA* or Δ*dps*Δ*cbpA* derivatives. Transformants were grown in LB medium at 37°C for three days. Plasmid DNA samples were prepared using a QIAprep Spin Miniprep Kit (Qiagen). Topoisomers were separated on a 1% agarose gel containing 2.5 µg/ml chloroquine. Gels were run for 60 hours at 40 V in the dark. After washing with water for at least 2 hours the gel was stained with ethidium bromide for 2 hours and photographed under UV illumination.

### Western blotting

Overnight cultures of *E. coli* BW25113 were diluted 1∶100 into fresh LB media and grown at 37°C with aeration. Samples (1 ml) were taken at the indicated OD_650_ values and the cells harvested by centrifugation. Cells were re-suspended in Laemmli buffer so that the number of cells in each sample was equivalent. After boiling for 10 min cytoplasmic proteins were separated by SDS-PAGE and Fis was detected using Western immunoblotting as previously described [43]. The blots were also probed using antibodies against RpoA (Neoclone) as a control.

## Supporting Information

Figure S1 Binding of Fis to DNA fragments carrying regulatory DNA sequences for genes encoding different nucleoid proteins. 175 nM, 350 nM or 700 nM Fis was incubated with different radiolabelled DNA fragments (∼20 nM) and the resulting protein-DNA complexes were separated by PAGE. Free DNA (F) and Fis-DNA complexes (C) are indicated. Promoters not known to contain Fis sites are shown in part A and panel B shows data for promoters with previously identified Fis binding sites. The *nirB* DNA fragment was included as a positive control.
(PDF)

Figure S2 *cbpA* regulatory region DNA fragments. The figure shows DNA sequences for *cbpA* regulatory region DNA fragments. The P6, P4, P1 and P2 promoters and transcription start sites are highlighted in purple, orange, red and blue respectively. Coding DNA sequences for the *yccE* and *cbpA* genes are shown in upper case. The Fis binding site between position −108 and −94 with respect to the P1 promoter is underlined. Note that the −10 hexamers for the P1 and P2 promoters overlap by 1 base pair. Mutations in different promoter elements are highlighted by a star.
(PDF)

Figure S3 Effects of mutations in primary Fis binding sequence. Results of an EMSA showing binding of Fis (150 nM, 225 nM, 300 nM, 450 nM or 600 nM) to the *cbpA* regulatory DNA (A) and a fragment carrying mutations in the putative Fis binding site (B). The DNA fragment was used present at a concentration of ∼20 nM.
(PDF)

Figure S4 Further characterisation of the *cbpA* P4 promoter. Panel A shows different P4*::lacZ* fusions. The P4 promoter is illustrated by an orange arrow. The Fis binding element is shown as a yellow bar. In the P4 −10con construct the P4 promoter −10 hexamer has been improved from the wild type 5′-TAAAAT-3′ sequence to 5′-TATAAT-3′. Panel B shows LacZ activity values for each promoter*::lacZ* fusion in growing JCB387 cells. We confirmed that the P4 and P4 −10con promoters produced transcripts of the same length (i.e. that we had not inadvertently created a new promoter in P4 −10con) using *in vitro* transcription assays. Thus, DNA fragments carrying P4 or P4 −10con were cloned in plasmid pSR and used as templates for *in vitro* transcription. The repressive effect of Fis on P4 was also confirmed in these experiments (Panel C).
(PDF)

Figure S5 Binding of Fis and transcription at the *cbpA* locus *in vivo*. The figure shows data from a ChIP-seq experiment to measure Fis binding across the *E. coli* chromosome (24) and an RNA-seq experiment using mRNA extracted from growing and stationary phase *E. coli* cells. The Fis binding profile is illustrated as a yellow line and reads mapping to different locations in the RNA-seq experiment are shown by arrows.
(PDF)

Figure S6 Chromosome-wide distribution of CbpA in starved *E. coli*+/−CbpM. Genome-wide view of CbpA binding in starved BW27784 (WT) and MC108 (Δ*cbpM*) cells. The figure shows ChIP-chip data for CbpA binding plotted against features of the *E. coli* genome in the form of a genome atlas. The data have been averaged across a 100,000 base pair window. The four chromosomal macrodomains (MD) are labelled.
(PDF)

Figure S7 Binding of CbpA to different targets *in vitro*. A) Distribution of CbpA across the *E. coli* chromosome. Genome-wide view of CbpA binding in starved MC108 cells. The figure shows ChIP-chip data for CbpA binding plotted against features of the *E. coli* genome in the form of a genome atlas. The data have been averaged across a 100,000 base pair window. The four chromosomal macrodomains (MD) are labelled. Regions selected for *in vitro* binding assays are highlighted. B) CbpA binding to the *glpX*, *yabN* and *paaA* loci *in vitro*. The figure shows ethidium bromide gels on which DNA fragments corresponding to the different genomic loci have been run in the presence and absence of CbpA (1.25, 2.5 or 5.0 mM). Reactions contained 0.1 mM DNA.
(PDF)

Table S1 Strains, plasmids, and oligonucleotide sequences.
(DOCX)

Table S2 Raw array data.
(ZIP)

Table S3 Genome browser file.
(ZIP)

Table S4 *E. coli* genome file.
(ZIP)
