# Supplementary material for: E. coli Fis Protein Insulates the cbpA Gene from Uncontrolled Transcription
Source: PLoS Genet. 2013 Jan 17;9(1):e1003152. doi: 10.1371/journal.pgen.1003152 (PMC3547828; doi:10.1371/journal.pgen.1003152)
Supplement: Figure S1 — Binding of Fis to DNA fragments carrying regulatory DNA sequences for genes encoding different nucleoid proteins. 175 nM, 350 nM or 700 nM Fis was incubated with different radiolabelled DNA fragments (∼20 nM) and the resulting protein-DNA complexes were separated by PAGE. Free DNA (F) and Fis-DNA complexes (C) are indicated. Promoters not known to contain Fis sites are shown in part A and panel B shows data for promoters with previously identified Fis binding sites. The nirB DNA fragment was included as a positive control. (PDF) [file pgen.1003152.s001.pdf]

Figure S1

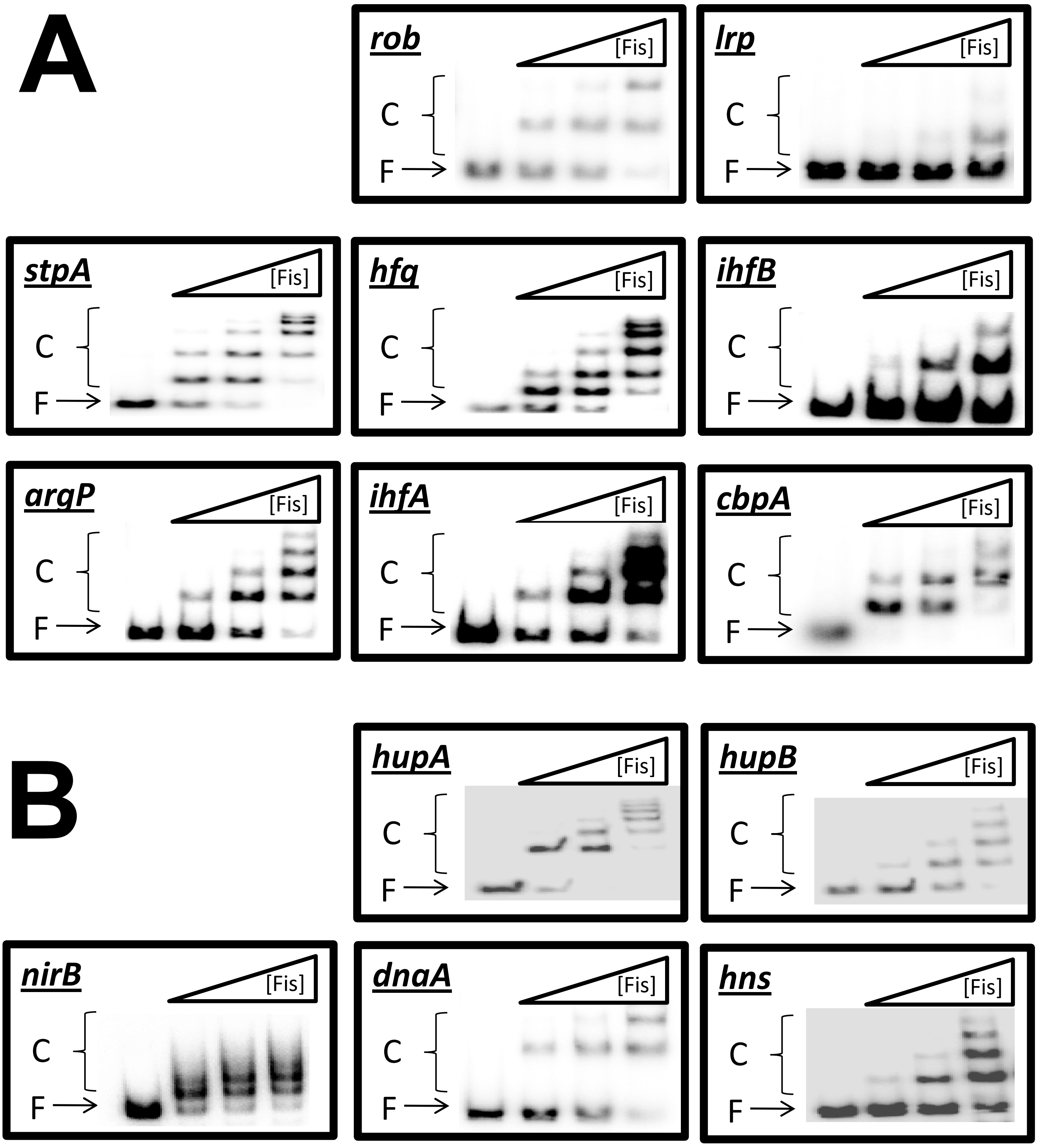

Figure S1: Binding of Fis to DNA fragments carrying regulatory DNA sequences for genes encoding different nucleoid proteins. 175 nM, 350 nM or 700 nM Fis was incubated with different radiolabelled DNA fragments and the resulting protein-DNA complexes were separated by PAGE. Free DNA (F) and Fis-DNA complexes (C) are indicated. Promoters not known to contain Fis sites are shown in part A and panel B shows data for promoters with previously identified Fis binding sites. The *nirB* DNA fragment was included as a positive control.
