# Supplementary material for: E. coli Fis Protein Insulates the cbpA Gene from Uncontrolled Transcription
Source: PLoS Genet. 2013 Jan 17;9(1):e1003152. doi: 10.1371/journal.pgen.1003152 (PMC3547828; doi:10.1371/journal.pgen.1003152)
Supplement: Figure S2 — cbpA regulatory region DNA fragments. The figure shows DNA sequences for cbpA regulatory region DNA fragments. The P6, P4, P1 and P2 promoters and transcription start sites are highlighted in purple, orange, red and blue respectively. Coding DNA sequences for the yccE and cbpA genes are shown in upper case. The Fis binding site between position −108 and −94 with respect to the P1 promoter is underlined. Note that the −10 hexamers for the P1 and P2 promoters overlap by 1 base pair. Mutations in different promoter elements are highlighted by a star. (PDF) [file pgen.1003152.s002.pdf]

Figure S2

Full length 302 base pair *cbpA* regulatory DNA

P6 transcript start
ATTTGCAGTGCAACTAATTCCATGTATATTACTACCCATatatagcgtctataaaatttaataaataa
-35 -10 *yccE* start codon

tgacgccctagttaaacttaaagtgcctggttcaactatcaaaaatcgctcacccttttttcacctgtt
-35 -108

P4 transcript start
taaaatatgttcagcaacccatcttgatggcgacctcctctccgcgatgatttcaataacatattctg
-10 -94

P1 & P2 transcript starts
tgttggcatatgaaattttgaggattaccctacacttataggagttaccttacaggggttccttcaat
-35 -35 -10 -10

ttgtgttgatttacgcgagataacgctATG
cbpA start codon

Full length 302 base pair *cbpA* regulatory with -94G and -108C mutations

P6 transcript start
ATTTGCAGTGCAACTAATTCCATGTATATTACTACCCATatatagcgtctataaaatttaataaataa
-35 -10 *yccE* start codon

*
tgacgccctagttaaacttaaagtgcctggttcaactatcaaaaatcgctcacccttttttcacctctt
-35 -108

* P4 transcript start
taaaatatgttgagcaacccatcttgatggcgacctcctctccgcgatgatttcaataacatattctg
-10 -94

P1 & P2 transcript starts
tgttggcatatgaaattttgaggattaccctacacttataggagttaccttacaggggttccttcaat
-35 -35 -10 -10

ttgtgttgatttacgcgagataacgctATG
cbpA start codon

Full length 302 base pair *cbpA* regulatory DNA with -217G and -216G mutations

```
                                      **      P6 transcript start
ATTTGCAGTGCAACTAATTCCATGGGTATTACTACCCATatatagcgtctataaaatttaataaataa
  -35                                   -10     yccE start codon

tgacgccctagttaaacttaaagtgcctggttcaactatcaaaaatcgctcacccttttttcacctgtt
                                                 -35            -108

           P4 transcript start
taaaatatgttcagcaacccatcttgatggcgacctcctctccgcgatgatttcaataacatattctg
 -10      -94

                                              P1 & P2 transcript starts
tgttggcatatgaaattttgaggattaccctacacttatagggagttaccttacaggggttccttcaat
   -35     -35                    -10  -10

ttgtgttgatttacgcgagataacgctATG
                        cbpA start codon
```

Full length 302 base pair *cbpA* regulatory DNA with -11G, -7G and -6G mutations

```
                                              P6 transcript start
ATTTGCAGTGCAACTAATTCCATGTATATTACTACCCATatatagcgtctataaaatttaataaataa
  -35                                   -10     yccE start codon

tgacgccctagttaaacttaaagtgcctggttcaactatcaaaaatcgctcacccttttttcacctgtt
                                                 -35            -108

           P4 transcript start
taaaatatgttcagcaacccatcttgatggcgacctcctctccgcgatgatttcaataacatattctg
 -10      -94

                                       *     **      P1 & P2 transcript starts
tgttggcatatgaaattttgaggattgccctggacttatagggagttaccttacaggggttccttcaat
   -35     -35                    -10  -10

ttgtgttgatttacgcgagataacgctATG
                        cbpA start codon
```

***cbpA* P4 fragment** (contains a 93 bp deletion between positions -94 and +1)

```
tcaactatcaaaaatcgctcacccttttttcacctgtttaaaatatgttcatagggagttaccttacagg
                -35                -108  -10       -94

ggttccttcaatttgtgttgatttacgcgagataacgctATG
                                    cbpA start codon
```

cbpA P4 -10con fragment

*

tcaactatcaaaaatcgctcaccctttttcacctgtttataatatgttcataggagttaccttacagg
-35 -108 -10 -94

ggttccttcaatttgtgttgatttacgcgagataacgct**ATG**
cbpA start codon

cbpA P6 fragment

P6 transcript start
ATTTGCAGTGCAACTAATTCCATGTATATTACTACCCATatatagcgtctataaaatttaataaataa
-35 -10 *yccE* start codon

tgacgccctagttaaacttaaagtgcctggttcaactatcaaaaatcgctcaccctttttcacctgtt
-35 -108

taaaatatgttcataggagttaccttacaggggttccttcaatttgtgttgatttacgcgagataacg
-10 -94
ct**ATG**
cbpA start codon

cbpA P6 Δ10

P6 transcript start
ATTTGCAGTGCAACTAATTCCATGTATATTACTACCCATataaaatttaataaataatgacgccctag
-35 -10 *yccE* start codon

ttaaacttaaagtgcctggttcaactatcaaaaatcgctcaccctttttcacctgtttaaaatatgtt
-35 -108 -10

cataggagttaccttacaggggttccttcaatttgtgttgatttacgcgagataacgct**ATG**
-94 *cbpA* start codon

cbpA P6 Δ20

P6 transcript start
ATTTGCAGTGCAACTAATTCCATGTATATTACTACCCATataaataatgacgccctagttaaacttaa
-35 -10 *yccE* start codon

agtgcctggttcaactatcaaaaatcgctcaccctttttcacctgtttaaaatatgttcataggagtt
-35 -108 -10 -94

accttacaggggttccttcaatttgtgttgatttacgcgagataacgct**ATG**
cbpA start codon

cbpA P6 Δ30

P6 transcript start
ATTTGCAGTGCAACTAATTCCATGTATATTACTACCCATacgccctagttaaacttaaagtgcctggt
-35 -10 *yccE* start codon

tcaactatcaaaaatcgctcaccctttttcacctgtttaaaatatgttcataggagttaccttacagg
-35 -108 -10 -94

ggttccttcaatttgtgttgatttacgcgagataacgct**ATG**
cbpA start codon

***cbpA* P6 Δ60**

P6 transcript start

ATTTGCAGTGCAACTAATTCCATGTATATTACTACCCATcaactatcaaaaatcgctcaccctttttcacctgtttaaaatatgttcataggagttaccttacaggggttccttcaatttgtgttgatttacgcgagataacgct**ATG**

-35 -10 *yccE* start codon -35 -108 -10 -94

cbpA start codon

***cbpA* P6 Δ80**

P6 transcript start

ATTTGCAGTGCAACTAATTCCATGTATATTACTACCCATcccttttttcacctgtttaaaatatgttcataggagttaccttacaggggttccttcaatttgtgttgatttacgcgagataacgct**ATG**

-35 -10 *yccE* start codon -108 -10 -94

cbpA start codon

***cbpA* P6 Δ100**

P6 transcript start

ATTTGCAGTGCAACTAATTCCATGTATATTACTACCCATatatgttcataggagttaccttacaggggttccttcaatttgtgttgatttacgcgagataacgct**ATG**

-35 -10 *yccE* start codon -94

cbpA start codon

Figure S2: *cbpA* regulatory region DNA fragments. The figure shows DNA sequences for *cbpA* regulatory region DNA fragments. The P6, P4, P1 and P2 promoters and transcription start sites are highlighted in purple, orange, red and blue respectively. Coding DNA sequences for the *yccE* and *cbpA* genes are shown in upper case. The Fis binding element between position -108 and -94 with respect to the P1 promoter is underlined. Note that the -10 hexamers for the P1 and P2 promoters overlap by 1 bp. Mutations in different promoter elements are highlighted by a star.
