# Supplementary material for: E. coli Fis Protein Insulates the cbpA Gene from Uncontrolled Transcription
Source: PLoS Genet. 2013 Jan 17;9(1):e1003152. doi: 10.1371/journal.pgen.1003152 (PMC3547828; doi:10.1371/journal.pgen.1003152)
Supplement: Figure S3 — Effects of mutations in primary Fis binding sequence. Results of an EMSA showing binding of Fis (150 nM, 225 nM, 300 nM, 450 nM or 600 nM) to the cbpA regulatory DNA (A) and a fragment carrying mutations in the putative Fis binding site (B). The DNA fragment was used present at a concentration of ∼20 nM. (PDF) [file pgen.1003152.s003.pdf]

Figure S3

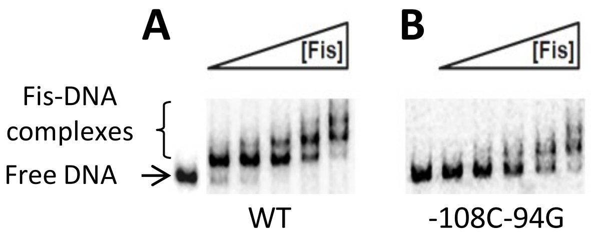

Figure S3: Effects of mutations in primary Fis binding sequence. Results of an EMSA showing reduced binding of Fis (150 nM, 225 nM, 300 nM, 450 nM or 600 nM) to the *cbpA* regulatory DNA (A) and a fragment carrying mutations in the putative Fis binding site (B). The DNA fragments were used at a concentration of 20 nM.
