# Supplementary material for: E. coli Fis Protein Insulates the cbpA Gene from Uncontrolled Transcription
Source: PLoS Genet. 2013 Jan 17;9(1):e1003152. doi: 10.1371/journal.pgen.1003152 (PMC3547828; doi:10.1371/journal.pgen.1003152)
Supplement: Figure S4 — Further characterisation of the cbpA P4 promoter. Panel A shows different P4::lacZ fusions. The P4 promoter is illustrated by an orange arrow. The Fis binding element is shown as a yellow bar. In the P4 −10con construct the P4 promoter −10 hexamer has been improved from the wild type 5′-TAAAAT-3′ sequence to 5′-TATAAT-3′. Panel B shows LacZ activity values for each promoter::lacZ fusion in growing JCB387 cells. We confirmed that the P4 and P4 −10con promoters produced transcripts of the same length (i.e. that we had not inadvertently created a new promoter in P4 −10con) using in vitro transcription assays. Thus, DNA fragments carrying P4 or P4 −10con were cloned in plasmid pSR and used as templates for in vitro transcription. The repressive effect of Fis on P4 was also confirmed in these experiments (Panel C). (PDF) [file pgen.1003152.s004.pdf]

Figure S4

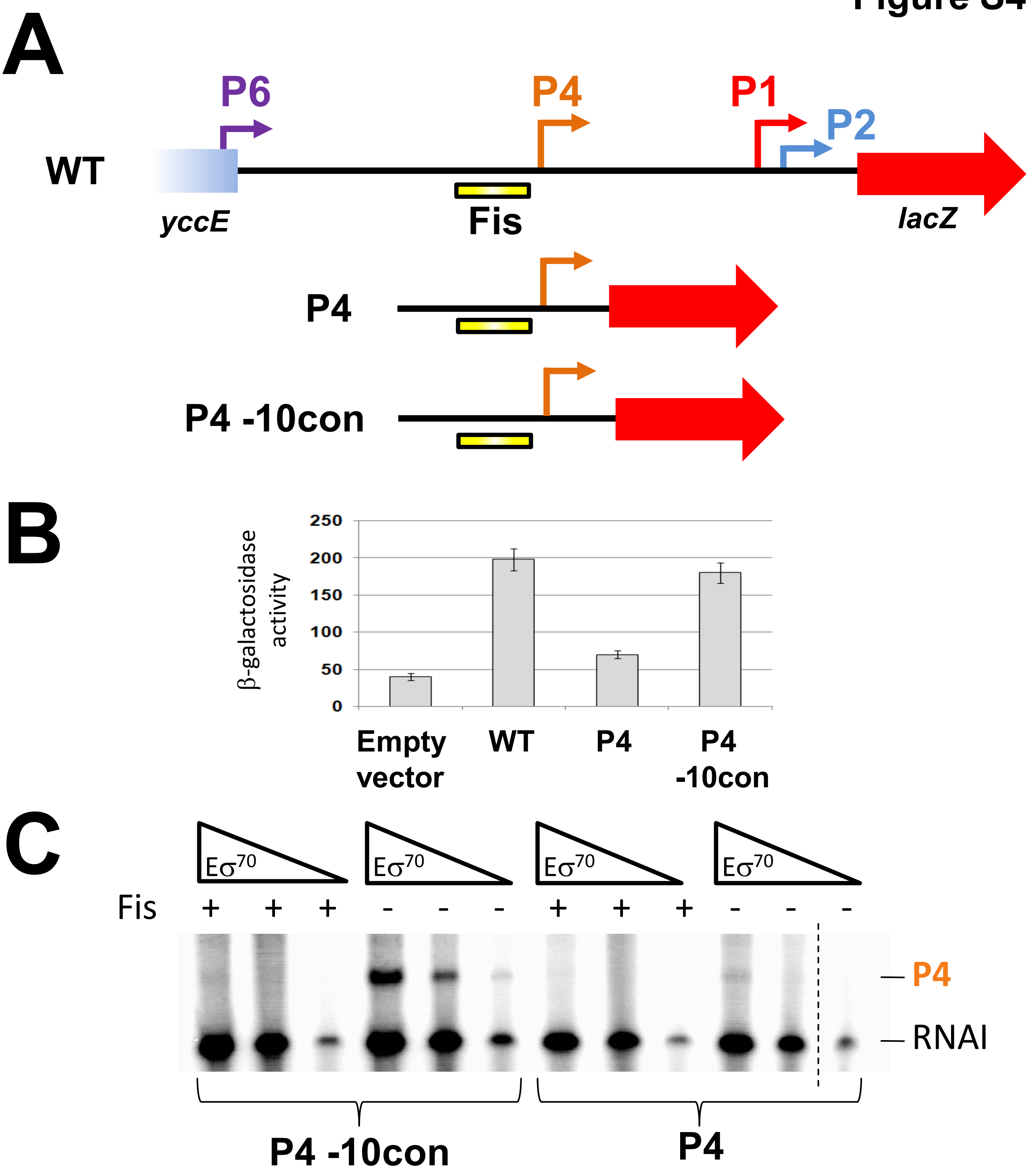

Figure S4: Further characterisation of the *cbpA* P4 promoter. Panel A shows different P4::*lacZ* fusions. The P4 promoter is illustrated by an orange arrow. The Fis binding element is shown as a yellow bar. In the P4 -10con construct the P4 promoter -10 hexamer has been improved from the wild type 5'-TAAAAT-3' sequence to 5'-TATAAT-3'. Panel B shows LacZ activity values for each promoter::*lacZ* fusion from growing JCB387 cells. We confirmed that the P4 and P4 -10con promoters produced transcripts of the same length using *in vitro* transcription assays. Thus, DNA fragments carrying P4 or P4 -10con were cloned in plasmid pSR and used as templates for *in vitro* transcription. The repressive effect of Fis on P4 was also confirmed in these experiments (Panel C).
