# Supplementary material for: E. coli Fis Protein Insulates the cbpA Gene from Uncontrolled Transcription
Source: PLoS Genet. 2013 Jan 17;9(1):e1003152. doi: 10.1371/journal.pgen.1003152 (PMC3547828; doi:10.1371/journal.pgen.1003152)
Supplement: Figure S5 — Binding of Fis and transcription at the cbpA locus in vivo. The figure shows data from a ChIP-seq experiment to measure Fis binding across the E. coli chromosome (24) and an RNA-seq experiment using mRNA extracted from growing and stationary phase E. coli cells. The Fis binding profile is illustrated as a yellow line and reads mapping to different locations in the RNA-seq experiment are shown by arrows. (PDF) [file pgen.1003152.s005.pdf]

Figure S5

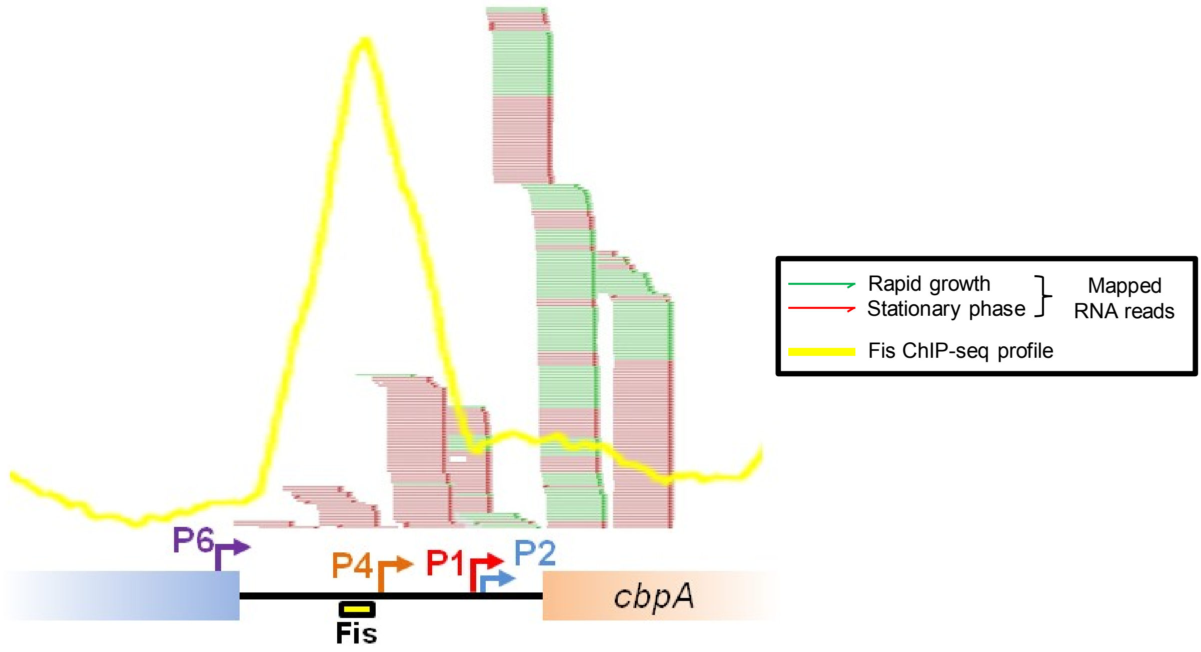

Figure S5: Binding of Fis and transcription at the *cbpA* locus *in vivo*. The figure shows data from a ChIP-seq experiment to measure Fis binding across the *E. coli* chromosome (24) and an RNA-seq experiment using mRNA extracted from growing and stationary phase *E. coli* cells. The Fis binding profile is illustrated as a yellow line and reads mapping to different locations in the RNA-seq experiment are shown by arrows.
