# Supplementary material for: E. coli Fis Protein Insulates the cbpA Gene from Uncontrolled Transcription
Source: PLoS Genet. 2013 Jan 17;9(1):e1003152. doi: 10.1371/journal.pgen.1003152 (PMC3547828; doi:10.1371/journal.pgen.1003152)
Supplement: Figure S6 — Chromosome-wide distribution of CbpA in starved E. coli+/−CbpM. Genome-wide view of CbpA binding in starved BW27784 (WT) and MC108 (ΔcbpM) cells. The figure shows ChIP-chip data for CbpA binding plotted against features of the E. coli genome in the form of a genome atlas. The data have been averaged across a 100,000 base pair window. The four chromosomal macrodomains (MD) are labelled. (PDF) [file pgen.1003152.s006.pdf]

Figure S6

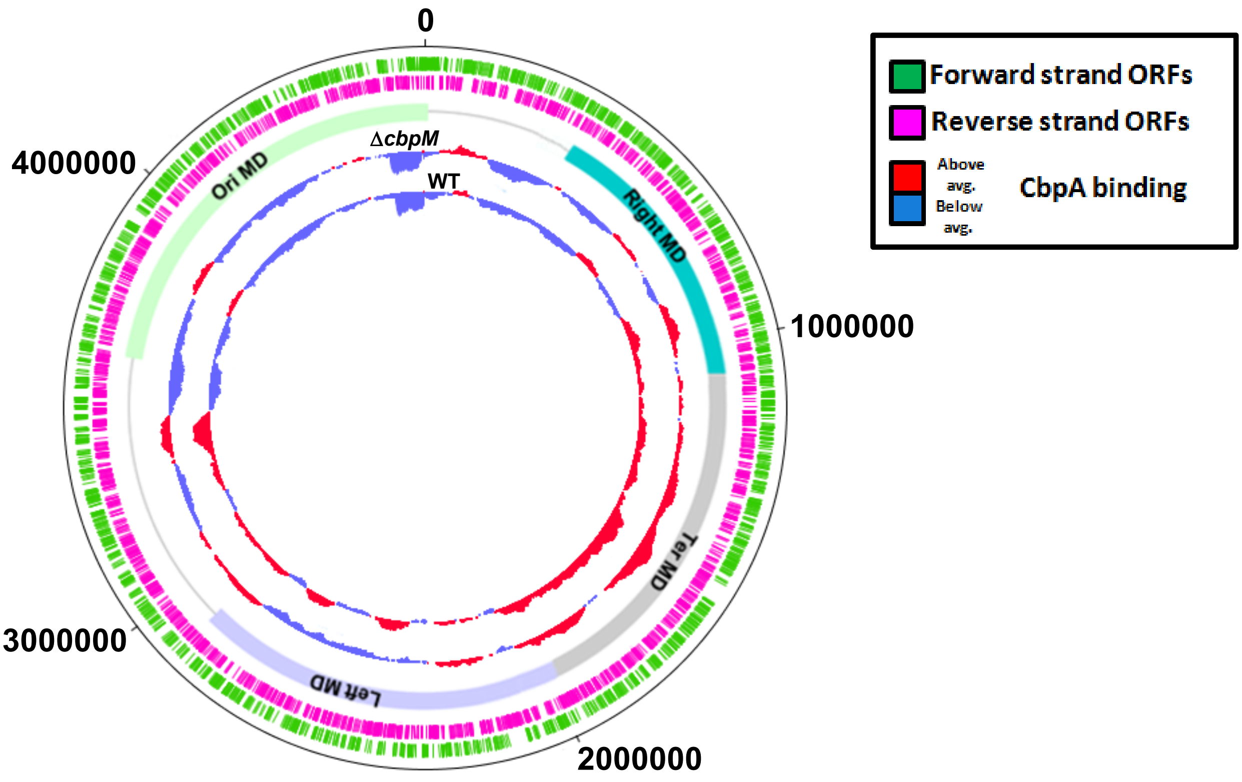

Figure S6: Chromosome-wide distribution of CbpA in starved *E. coli* +/- CbpM. Genome-wide view of CbpA binding in starved BW27784 (wt) and MC108 cells. The figure shows ChIP-chip data for CbpA binding plotted against features of the *E. coli* genome in the form of a genome atlas. The data have been averaged across a 100,000 base pair window. The four chromosomal macrodomains (MD) are labelled.
