# Supplementary material for: E. coli Fis Protein Insulates the cbpA Gene from Uncontrolled Transcription
Source: PLoS Genet. 2013 Jan 17;9(1):e1003152. doi: 10.1371/journal.pgen.1003152 (PMC3547828; doi:10.1371/journal.pgen.1003152)
Supplement: Figure S7 — Binding of CbpA to different targets in vitro. A) Distribution of CbpA across the E. coli chromosome. Genome-wide view of CbpA binding in starved MC108 cells. The figure shows ChIP-chip data for CbpA binding plotted against features of the E. coli genome in the form of a genome atlas. The data have been averaged across a 100,000 base pair window. The four chromosomal macrodomains (MD) are labelled. Regions selected for in vitro binding assays are highlighted. B) CbpA binding to the glpX, yabN and paaA loci in vitro. The figure shows ethidium bromide gels on which DNA fragments corresponding to the different genomic loci have been run in the presence and absence of CbpA (1.25, 2.5 or 5.0 mM). Reactions contained 0.1 mM DNA. (PDF) [file pgen.1003152.s007.pdf]

Figure S7

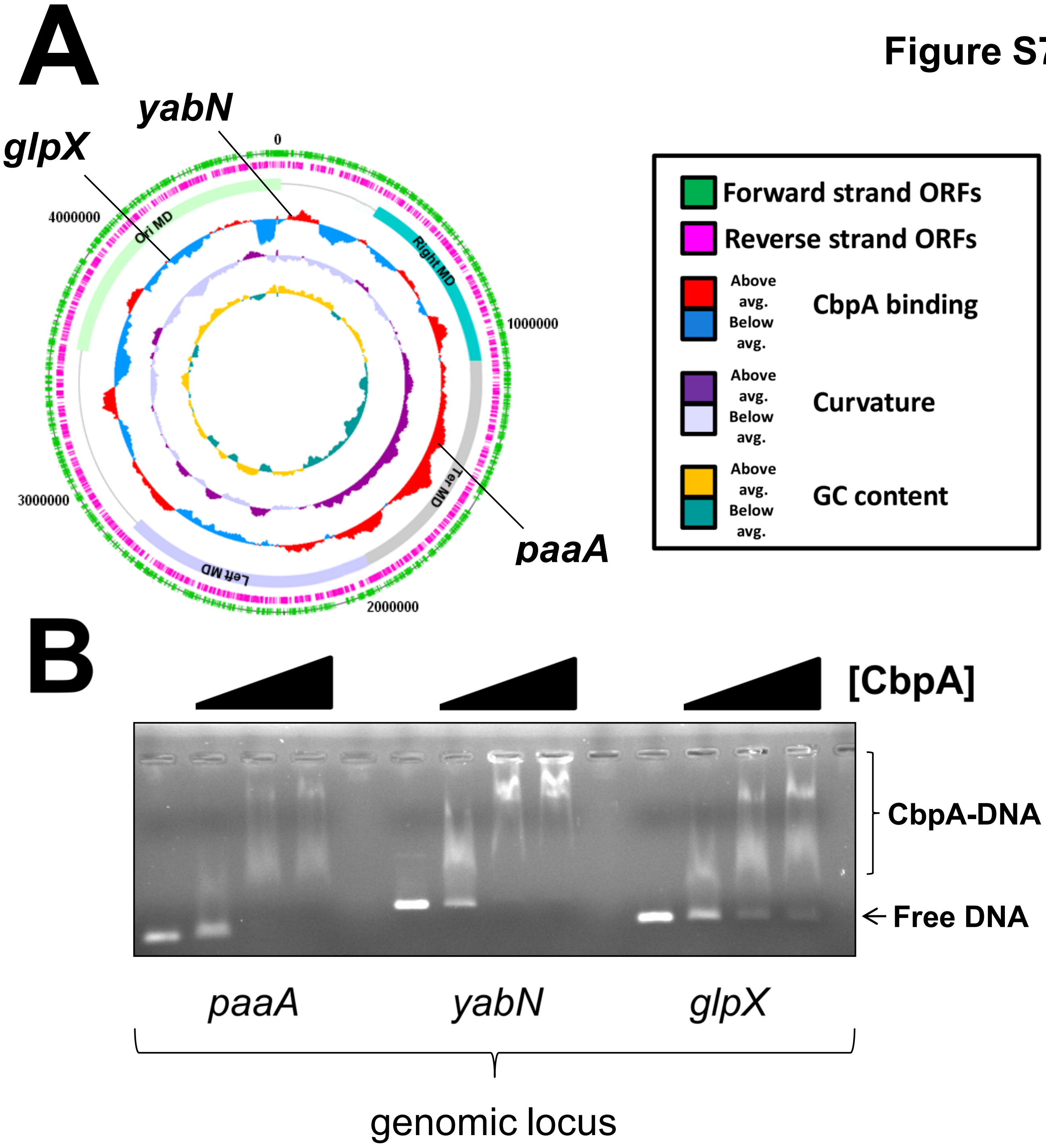

Figure S7: Binding of CbpA to different targets *in vitro*.

A) Distribution of CbpA across the *E. coli* chromosome. Genome-wide view of CbpA binding in starved MC108 cells. The figure shows ChIP-chip data for CbpA binding plotted against features of the *E. coli* genome in the form of a genome atlas. The data have been averaged across a 100,000 base pair window. The four chromosomal macrodomains (MD) are labelled. Regions selected for in vitro binding assays are highlighted.

B) CbpA binding to the *glpX*, *yabN* and *paaA* loci *in vitro*. The figure shows ethidium bromide gels on which DNA fragments corresponding to the different genomic loci have been run in the presence and absence of CbpA (2.5, 3.75 or 5 μM). Reactions contained 1 μM DNA.
