# Supplementary material for: E. coli Fis Protein Insulates the cbpA Gene from Uncontrolled Transcription
Source: PLoS Genet. 2013 Jan 17;9(1):e1003152. doi: 10.1371/journal.pgen.1003152 (PMC3547828; doi:10.1371/journal.pgen.1003152)
Supplement: Table S1 — Strains, plasmids, and oligonucleotide sequences. (DOCX) [file pgen.1003152.s008.docx]

**Table S1: Strains and plasmids**

| **Name** | **Description** | **Source** |
| --- | --- | --- |
| **Bacterial strains** | | |
| JCB387 | Δ*nir* Δ*lac* | Page *et. al*. (1990) |
| JCB3871Δ*fis* | (JCB387) *fis985* (*str/spcR*) | Wu *et. al*. (1998) |
| BTH101 | F′ cya-99 araD139 galE15 galK16 rpsL1(StrR) hsdR2 mcrA1 mcrB1 | Karimova et al. (2001) |
| T7 express | fhuA2 lacZ::T7 gene1 [lon] ompT gal sulA11 R(mcr-73::miniTn10–TetS)2[dcm] R(zgb-210::Tn10–TetS) endA1 D(mcrC-mrr)114::IS10 | (Invitrogen) |
| MC108 | (BW27784) ΔcbpM3::cat | Chenoweth *et al*. (2007) |
| BW27784 | (BW25113) DE(araFGH) (ΔaraEp PCP18-araE) | Khlebnikov et al. (2001) |
| MC105 (BW27784) | ΔcbpA3::cat | Chenoweth *et al*. (2007) |
| KC101 | (BW27784) *dps*::kan | P1 transduction from JW0797 This study |
| KC102 | (MC105) *dps*::kan | P1 transduction from JW0797 This study |
| JW0797 | *dps*::kan | Yamamoto *et al*. (2009) |
| **Bacterial plasmids** | | |
| pRW50 | Broad-host-range *lac* fusion vector for cloning promoters on *Eco*RI–*Hin*dIII fragments: contains the RK2 origin of replication and encodes TcR | Lodge *et. al*. (1992) |
| pSR | pBR322-derived plasmid containing an *Eco*RI–*Hin*dIII fragment upstream of the λ*oop* transcription terminator | Kolb *et. al*. (1995) |
| pET21a | T7 Expression vector containing 6xHis tag | (Novagen) |
| pJ204 | pUC derivative encoding AmpR | (DNA2.0) |
| **Oligonucleotides** (all sequences in 5’-3’ direction) | | |
| *cbpA* up | ggctgcgaattctatttgcagtgcaactaattccatg | This work. Used with cbpA down to amplify the *cbpA* regulatory region from the *E. coli* K-12 chromosome. |
| *cbpA* down | cgcccgaagcttcatagcgttatctcgcgtaaatc | This work |
| *cbpA* -108C-94G | gctcaccctttttcacctctttaaaatatgttgagcaacccatcttg | This work. Used to mutate the primary *cbpA* Fis binging site |
| *cbpA* Δ45 | ggctgcgaattcatattctgtgttggcatatg | This work. Used to generate the *cbpA* promoter Δ45 fragment |
| *cbpA* Δ93 up | ggctgcgaattcaaaaatcgctcaccctttttcacctgtttaaaatatgttcataggagttaccttacaggggttccttc | This work. Used with *cbpA* Δ93 down to make the *cbpA* Δ93 promoter fragment. |
| *cbpA* Δ93-10con up | ggctgcgaattcaaaaatcgctcaccctttttcacctgtttataatatgttcataggagttaccttacaggggttccttc | This work. Used with *cbpA* Δ93 down to make the *cbpA* Δ93 -10con promoter fragment. |
| *cbpA* Δ93 down | cgcccgaagcttcatagcgttatctcgcgtaaatcaacacaaattgaaggaacccctgtaaggtaactcctatgaacata | This work. |
| *cbpA* Δ203 up | ggctgcgaattctatttgcagtgcaactaattccatgtatattactacccataggagttaccttac | This work. Used with cbpA D203 down or derivatives to make *cbpA*P6 *lacZ* fusions (Figure 4A). |
| *cbpA* Δ203 down | cgcccgaagcttcatagcgttatctcgcgtaaatcaacacaaattgaaggaacccctgtaaggtaactcctactgaacatattttaaacaaggtgaaaaa | This work. |
| *cbpA* Δ203 up Δ10 | ggctgcgaattctatttgcagtgcaactaattccatgtatattactacccatataaaatttaataaataa | This work. |
| *cbpA* Δ203 up Δ20 | ggctgcgaattctatttgcagtgcaactaattccatgtatattactacccatataaataatgacgccctagttaaacttaaagtg | This work. |
| *cbpA* Δ203 up Δ30 | ggctgcgaattctatttgcagtgcaactaattccatgtatattactacccatacgccctagttaaacttaaagtgcctggttc | This work. |
| *cbpA* Δ203 up Δ60 | ggctgcgaattctatttgcagtgcaactaattccatgtatattactacccatcaactatcaaaaatcgctcaccctttttcacctgtt | This work. |
| *cbpA* Δ203 up Δ80 | ggctgcgaattctatttgcagtgcaactaattccatgtatattactacccatccctttttcacctgtttaaaatatgttcag | This work. |
| *cbpA* Δ203 up Δ100 | ggctgcgaattctatttgcagtgcaactaattccatgtatattactacccatatatgttcagaggagttaccttacagggg | This work. |
| *cbpA* up -216G-217G | ggctgcgaattctatttgcagtgcaactaattccatgggtattactacccatatatag | This work. Derivative of *cbpA* up. |
| *cbpA* down -11G-7G-6G | cgcccgaagcttcatagcgttatctcgcgtaaatcaacacaaattgaaggaacccctgtaaggtaactcctataagtgccgggcaatcctcaaaatttc | This work. Derivative of *cbpA* down. |
| *gal*P1 up + Fis binding region | ggctgcgaattccactaatttattccatgtcacacttttcgcatcttttttatgctatggttatttcatacgccctagttaaacttaaagtgcctggttc | This work. |
| *gal*P1 up - Fis binding region | ggctgcgaattcttgtgtaaacgattccactaatttattccatgtcacacttttcgcatcttttttatgctatggttatttca | This work. |
| *gal*P1 down | cgcccgaagctttgaaataaccatagcataaaaaagatgc | This work. Used with *gal*P1 up derivative. |
| *aer* up + Fis binding region | ctgcgaattctgcgatctaaatcaaattaatcggttaaagataaccgcagcggggccgacataaactctacgccctagttaaacttaaagtgcctggttc | This work. |
| *aer* up - Fis binding region | ggctgcgaattcttaaatcgcaaattgcgatctaaatcaaattaatcggttaaagataaccgcagcggggccgacataaactc | This work. |
| *aer* down | cgcccgaagcttgagtttatgtcggccccgctgcg | This work. Used with *aer* up derivative. |
| *yeaR* up + Fis binding region | ggctgcgaattcaaagtaaccaataaatggtatttaaaatgcaaattatcaggcgtaccctgaaacggtacgccctagttaaacttaaagtgcctggttc | This work. |
| *yeaR* up - Fis binding region | ggctgcgaattcgctaaaaagtaaccaataaatggtatttaaaatgcaaattatcaggcgtaccctgaaacgg | This work. |
| *yeaR* down | cgcccgaagcttccgtttcagggtacgcctgat | This work. Used with *yeaR* up derivative. |
| *ynfE* up + Fis binding region | ggctgcgaattccccttctattgatatggattaataattcttaacccaaaatgggtagactccctctatacgccctagttaaacttaaagtgcctggttc | This work. |
| *ynfE* up - Fis binding region | ggctgcgaattctcatacccttctattgatatggattaataattcttaacccaaaatgggtagactccctcta | This work. |
| *ynfE* down | cgcccgaagctttagagggagtctacccattttg | This work. Used with *ynfE* up derivative. |

**REFERENCES**

32. Page L, Griffiths L, Cole JA. (1990) Different physiological roles of two independent pathways for nitrite reduction to ammonia by enteric bacteria. *Arch Microbiol*. **154:**349-54.

Wu H, Tyson KL, Cole JA, Busby SJ. (1998) *Regulation of transcription initiation at the Escherichia coli nir operon promoter: a new mechanism to account for co-dependence on two transcription factors. Mol Microbiol.* **27:**493-505.

Chenoweth MR, Trun N, Wickner S. (2007) In vivo modulation of a DnaJ homolog, CbpA, by CbpM. *J Bacteriol.* **189:**3635-8.

Khlebnikov A, Datsenko KA, Skaug T, Wanner BL, Keasling JD. (2001) Homogeneous expression of the P(BAD) promoter in Escherichia coli by constitutive expression of the low-affinity high-capacity AraE transporter. *Microbiology.* **147:**3241-7.

Yamamoto N, Nakahigashi K, Nakamichi T, Yoshino M, Takai Y, Touda Y, Furubayashi A, Kinjyo S, Dose H, Hasegawa M, Datsenko KA, Nakayashiki T, Tomita M, Wanner BL, Mori H. (2009) Update on the Keio collection of Escherichia coli single-gene deletion mutants. *Mol Syst Biol.* **5:**335.

Lodge J, Fear J, Busby S, Gunasekaran P, Kamini NR. (1992) Broad host range plasmids carrying the Escherichia coli lactose and galactose operons. *FEMS Microbiol Lett.* **74:**271-6.

Kolb A, Kotlarz D, Kusano S, Ishihama A. (1995) Selectivity of the Escherichia coli RNA polymerase E sigma 38 for overlapping promoters and ability to support CRP activation. *Nucleic Acids Res*. **23:**819-26.
